# Supplementary material for: Interacting humans use forces in specific frequencies to exchange information by touch
Source: Sci Rep. 2022 Sep 21;12:15752. doi: 10.1038/s41598-022-19500-1 (PMC9492785; doi:10.1038/s41598-022-19500-1)
Supplement: Supplementary file 1 — Supplementary Information. [file 41598_2022_19500_MOESM1_ESM.pdf]

Supplementary

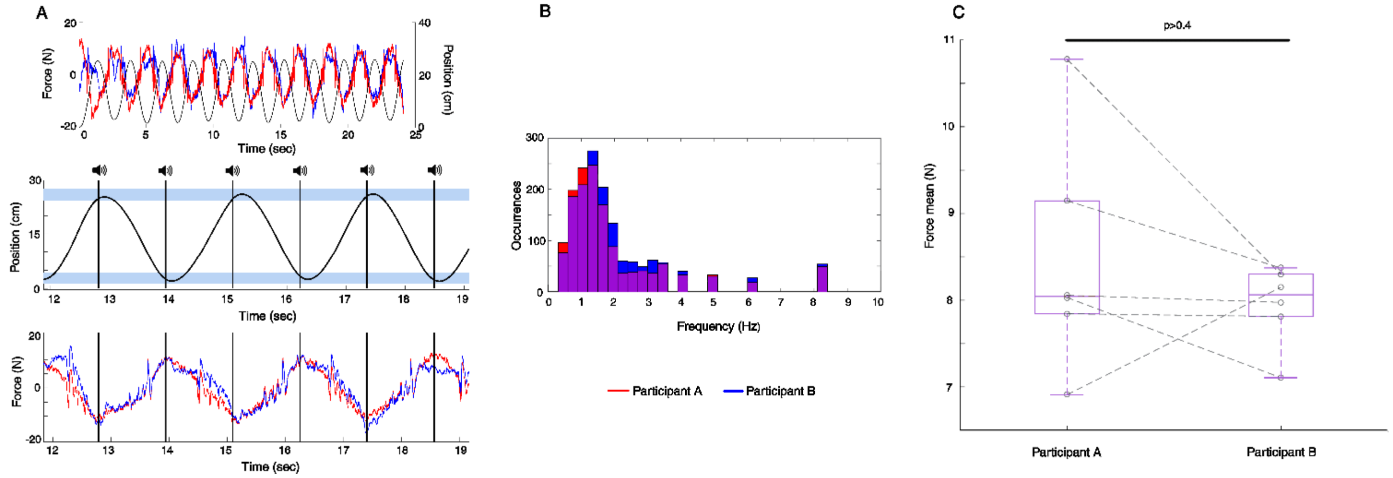

Fig. S1) Forces time series and task behavior in a representative trial for experiment 2, histograms of periodic components in forces, and force amplitudes statistics. A) Example of recorded displacement of the manipulandum (top) and forces (bottom) from a representative dyad in Experiment 2. Temporal cues as heard by the Synch Participant are represented as vertical black lines, and target area as horizontal pale bands. Participants were required to synchronize their change of direction with the metronome, while stopping at each target as accurately as possible**. B)** Histograms of frequency content of the participant's forces' time series. **C)** Average and individuals applied force by each participant in each dyad, across all conditions. We found no difference (p>0.4, T(10)=0.88) as confirmed by a two-sample t-test.

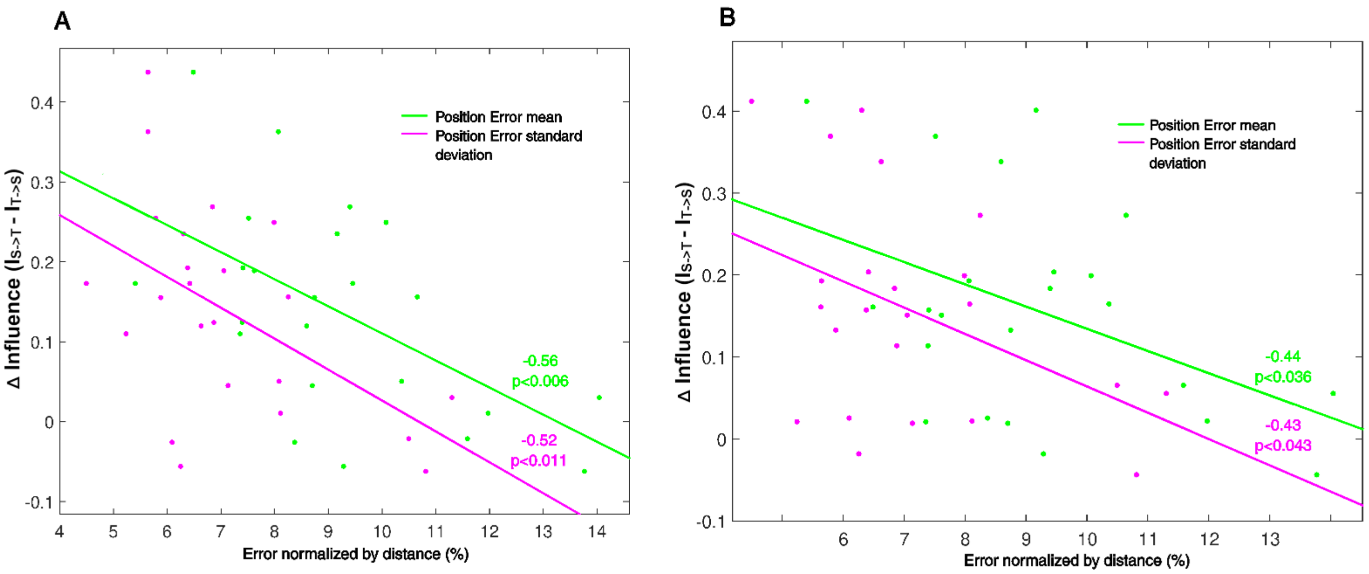

Fig. S2) Significant correlations of the Position Error performance in experiment 2. A) Negatives correlations of the Position Error mean and standard deviation with the delta Granger Causality in the [2.15-4] Hz frequency band (PEm p<0.006, PEsd p<0.01). **B)** Negatives correlations of the Position Error mean and standard deviation with the delta Granger Causality in the [4-6] Hz frequency band (PEm p<0.036, PEsd p<0.043).

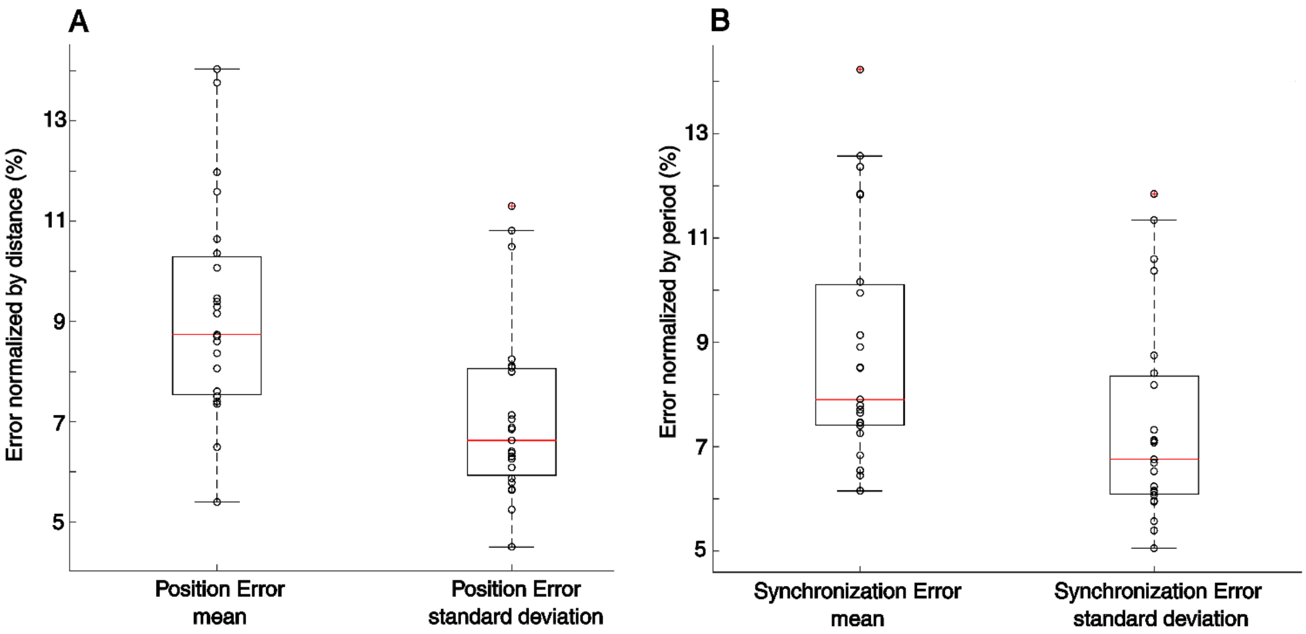

Fig. S3) Average and individuals error indices by each dyad, across all conditions in experiment 1. A) Average and individuals Position Error mean and Position Error standard deviation by each dyad, across all conditions. **B)** Average and individuals Synchronization Error mean and Synchronization Error standard deviation by each dyad, across all conditions.

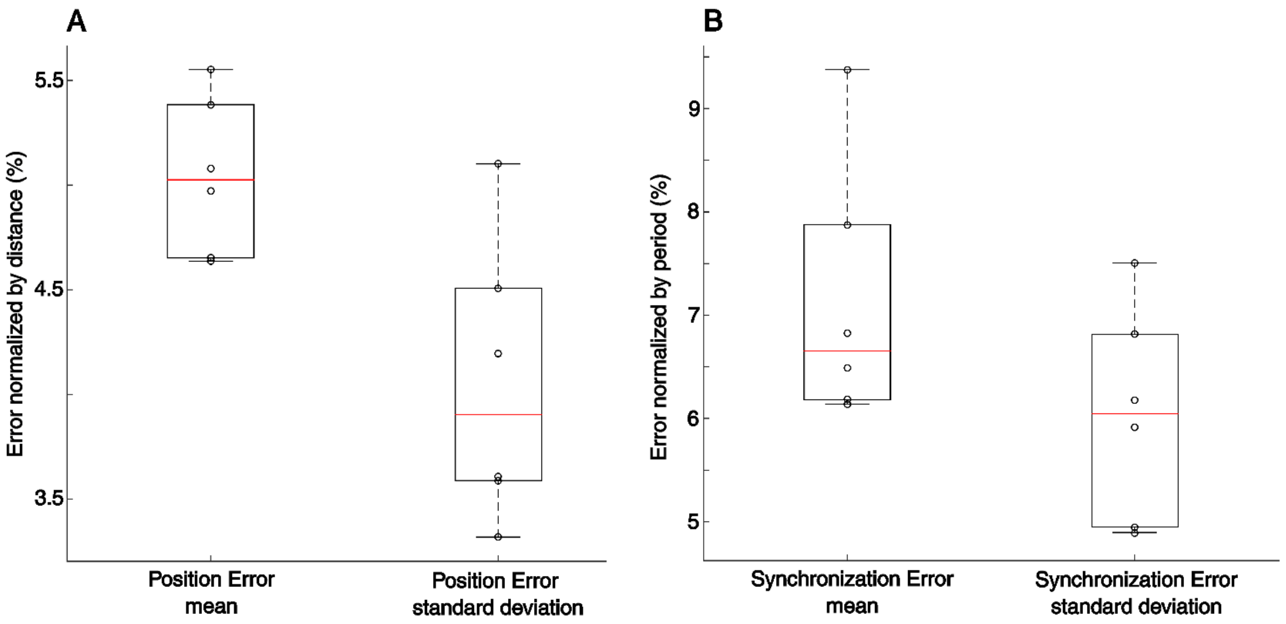

Fig. S4) Average and individuals error indices by each dyad, across all conditions in experiment 2. A) Average and individuals Position Error mean and Position Error standard deviation by each dyad, across all conditions. **B)** Average and individuals Synchronization Error mean and Synchronization Error standard deviation by each dyad, across all conditions.
